# The increasing burden of revision arthroplasty for infection and fracture: perspectives of a tertiary referral centre

**DOI:** 10.1177/11207000261469476

**Published:** 2026-09-02

**Authors:** Thomas Robertson, Oscar Kinman, Samuel H. Benveniste, Kerry Costi, Taisha D’Apollonio, Deepti Sharma, Kate Southam, Gerald Atkins, Michael Wyatt, Lucian Bogdan Solomon, Boopalan Ramasamy, Stuart A Callary

**Affiliations:** 1 Department of Orthopaedics and Trauma, Royal Adelaide Hospital, Adelaide, SA, Australia; 2 Discipline of Orthopaedics and Trauma, College of Health, Adelaide University, Adelaide, SA, Australia; 3 Lingard, Mayo and Forster Private Hospitals, NSW, Australia

**Keywords:** Arthroplasty, infection, revision, training

## Abstract

**Background:**

In public hospitals the number of complex urgent and semi-urgent revision joint replacements has increased over time, conversely the number of primary joint replacements has decreased. This study aimed to investigate the effect of this change on service delivery of elective surgery and training in one large metropolitan hospital.

**Methods:**

We retrospectively reviewed changes in staffing, workload and all hip and knee joint replacements performed at the Royal Adelaide Hospital over a 20-year period, between January 2003 to December 2023.

**Results:**

The number of primary total hip replacements (THR) and total knee replacements (TKR) performed on elective lists has decreased from 210/year to 140/year. The number of revisions THR and TKR increased from 76/year to 219/year (135 septic). The number of patients awaiting elective surgery has increased from 220 in 1999 (THR 18) to 597 in 2024 (THR 69).

**Conclusions:**

There is an urgent need of decisions by local policy makers to plan for the continual increase in THR and TKR as well as revision joint replacements. The significant change in pathology requires consideration of staffing, training and funding to account for the increased number of complex septic revisions.

## Introduction

Primary total hip (pTHR) and knee replacement (pTKR) surgeries are not only the most common orthopaedic elective procedures but also the most beneficial with respect to patient satisfaction and cost effectiveness.^1^ In Australia, 136,654 pTHR/pTKR were performed in 2023.^2^ These surgeries are also a fundamental component in the training of the next generation of orthopaedic surgeons.

The 25-year pooled survivorship of pTHR in cohort studies and joint registries is 77.6% and 57.9% respectively^3^ so many of these will come to require revision surgery. Therefore, as the number of pTHR and pTKR performed continues to rise^4^ so too has the demand for revision total hip (rTHR) and knee replacement (rTKR), with 9704 rTHR and rTKR procedures performed in Australia in 2023.^2^ The introduction of low wearing highly crosslinked polyethylene bearing implants in the early 2000s has reduced the proportion of revisions for wear and osteolysis,^5^ however the number of revisions for other causes, such as prosthetic joint infections (PJI) are on the rise.^2,6^ PJI is now the most common reason for performing a rTHR and rTKR in Australia,^2^ with approximately 4000 performed annually.^7^

PJI presents a range of particularly complex challenges for patients, health care systems, and society. The PIANO study demonstrated across 27 hospitals in Australia and New Zealand that 40% of PJIs are not cured within 2 years,^8^ and similar findings have been reported from Canada.^9,10^ According to the Australian Orthopaedic Association National Joint Replacement Registry (AOANJJR), >35% of patients undergoing revision for PJI require a subsequent revision within 1 year.^11^ Mortality following PJI diagnosis is substantial, reaching 20% at 5 years and 40% at 10 years, which is higher than rates associated with most common cancers.^12,13^ The quality of life of some patients with chronic PJI has been reported as ‘worse than death’.^14^ To improve outcomes, it is recommended that PJI are managed by multidisciplinary teams (MDT) in specialised centres.^15–17^ The hospital cost of PJI is expensive and for Australian public hospitals, using Australian Refined Diagnosis Related Groups (AR-DRG), was recently estimated to be AU$64,585 (SD AU$53,550).^18^

The AOANJJR reports that although overall the number of pTHR performed in Australia annually has continually increased over time, this is not the case for public hospitals, where the number have recently decreased.^11^ Decreases were first noticed during the COVID-19 pandemic, and the number of pTHRs performed in Australian public hospitals up to 2023 has remained stagnant whilst the number performed in private hospitals continues to increase (Figure 1(b)).^19^ The reduced number of pTHRs performed has coincided with a decrease in the number of public hospital beds relative to the population.^20^ For many Australian public hospitals, the waiting time for patients to be seen in outpatient clinics for ‘non-urgent’ conditions remains very long, sometimes years. Once eventually assessed in clinic, most elective orthopaedic conditions are deemed Category 3, or non-urgent, indicating that surgery should be performed within 1 calendar year of being placed on a waiting list. This commonly leads to the further deterioration of the condition which can severely affect patient quality of life. Unfortunately, 33% of patients awaiting to be seen in a typical orthopaedic outpatient clinic in Australia suffer from depression that is correlated to their pain, with 60% of these patients meeting the criteria for a major depressive disorder.^21^ For lower limb pathologies, prolonged delays to surgery can translate to inability to ambulate and a substantial increase in the support these patients require for their activities of daily living.

**Figure 1.**
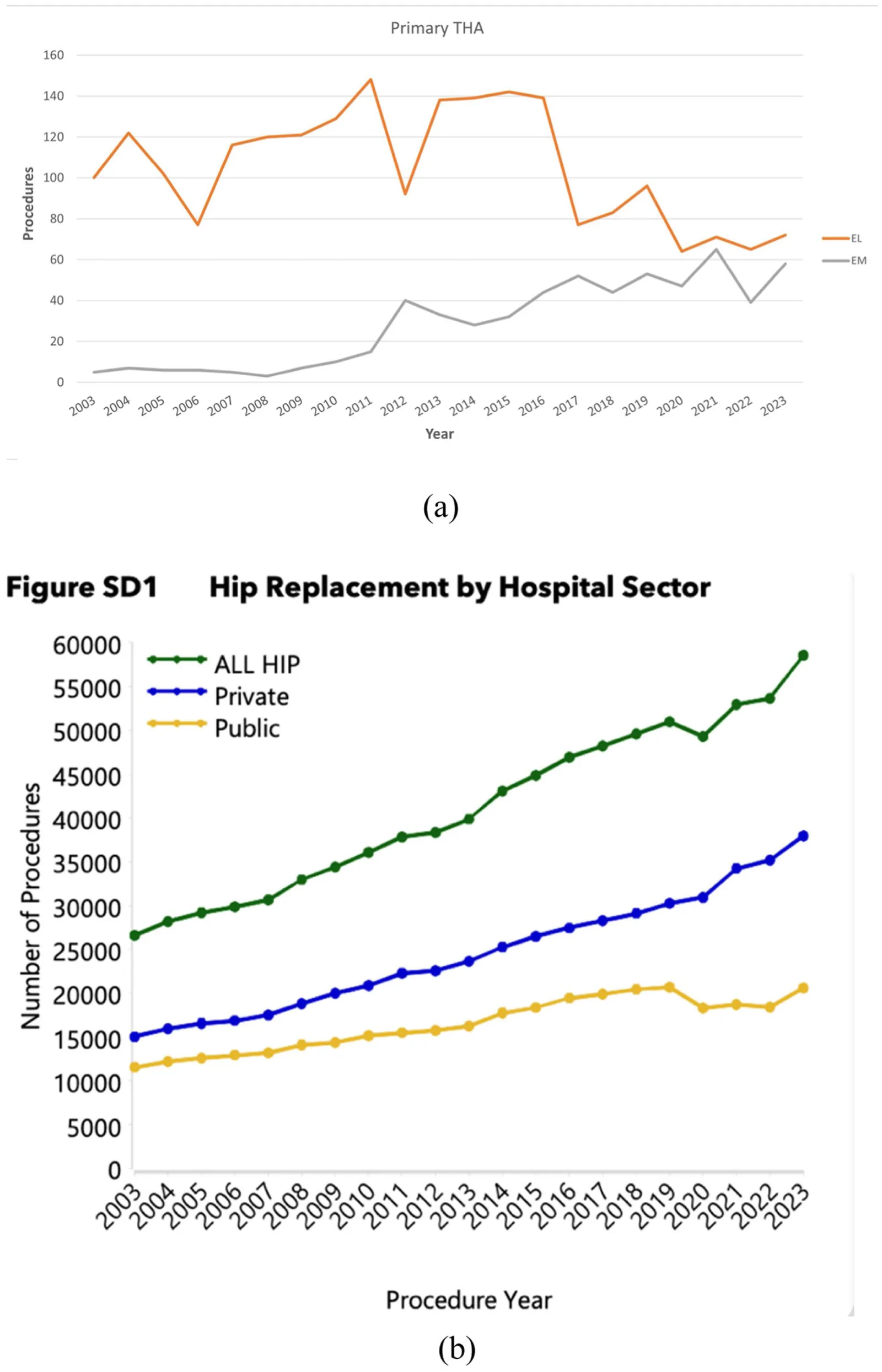
The number of elective and emergency (a) primary THR performed at the RAH since 2003 (b) Numbers of hip replacements performed by hospital sector in 2024 AOANJRR demographics of hip, knee and shoulder arthroplasty supplementary report.^19^

### The experience of a public tertiary referral centre

The Royal Adelaide Hospital (RAH) is the major trauma centre and is a tertiary referral centre, including for joint replacement matters, not only for the Adelaide metropolitan area but also serving regional South Australia, the Northern Territory, as well as parts of Victoria, New South Wales, Queensland and Western Australia. The hospital performs over 3000 orthopaedic surgeries every year. Over the last decade, there has been a trend of increasing emergency relative to elective orthopaedic surgeries (Figure 2(a)), with joint replacement related emergency procedures being of the most noticeable increase in pathology. In the last 5 years, the COVID-19 pandemic has had a long and lasting impact on the timely management of ‘less urgent’ medical conditions. A departmental review of this lasting impact on the elective surgery performed in our institution identified several other factors that affected our service delivery and prompted a root analysis of the problem to inform referrers and policy makers.

**Figure 2.**
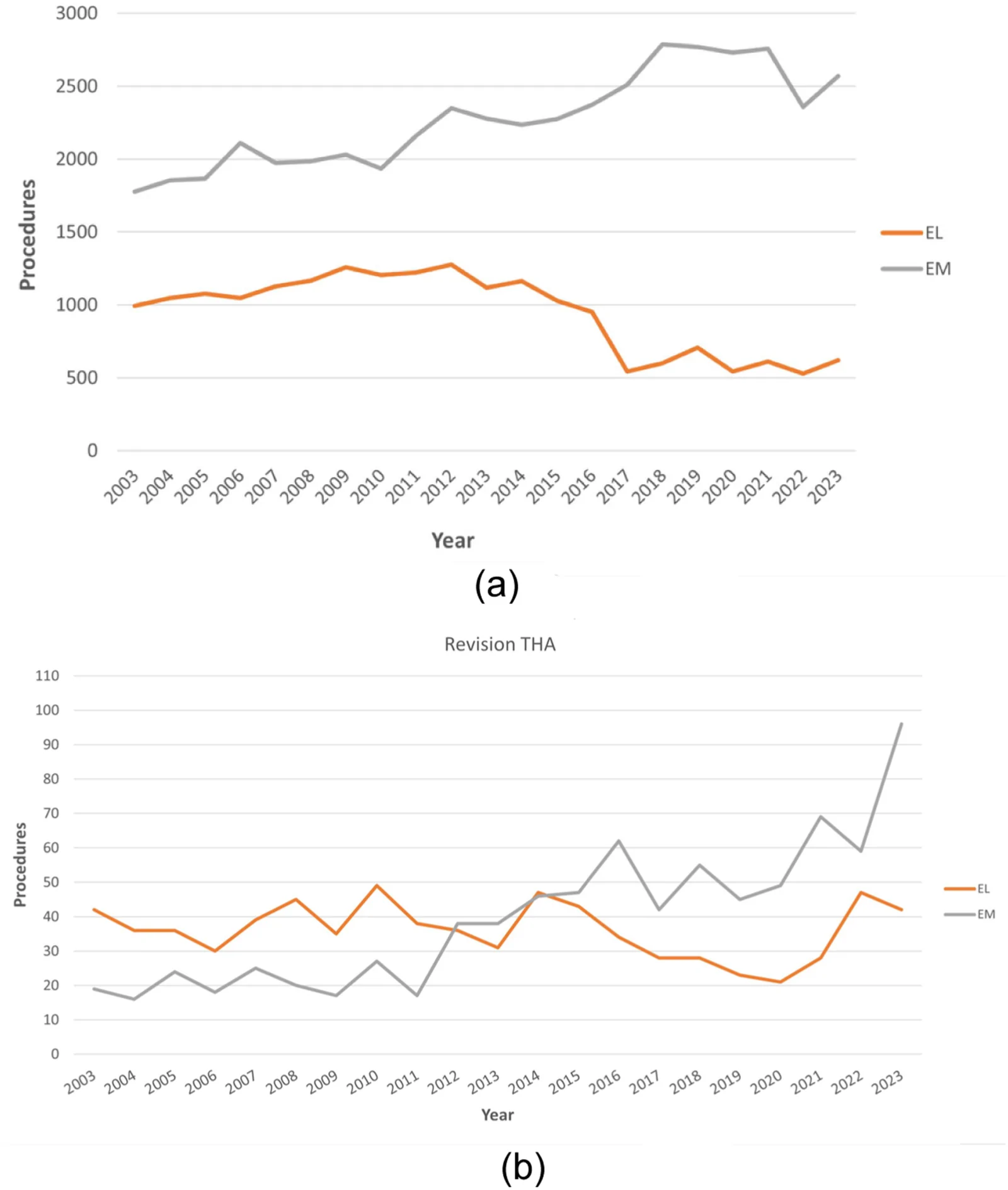
(a) The number of elective and emergency orthopaedic procedures performed at the RAH since 2003. (b) The number of elective and emergency revision THR performed at the RAH since 2003.

## Methods

Staffing levels over the 20 years between 2003–2023 were retrieved from administrative data. To establish the follow-on effect, the number of primary joint replacement cases for trainees and unaccredited registrars was sourced from the surgical procedure logs. Numbers of patients on surgical waitlist or awaiting OPD were taken from the regular audits undertaken by the joint replacement nurses.

All primary and revision THR and TKR performed at the RAH from January 2003 to December 2023 were retrospectively reviewed. Data were reviewed from the Orthopaedic Patient Management Outcomes Database (OPMOD), a prospective hospital-based joint replacement register, and theatre records from the Operating Room Management Information System (ORMIS). Revision surgery was defined as either component exchange or removal (debridement, antibiotics and implant retention (DAIR), single and 2-stage revisions surgeries).

To establish the current demands on orthopaedic theatres and wards resources, all PJI-related surgeries performed between January 2022 to December 2023 were extracted from electronic records in ORMIS and from OPMOD and then cross-referenced with the Joint Replacement Nurse. Each case was reviewed by the authors to assess if the case was undertaken in either an elective and emergency theatre and to identify the hospital of the index pTHR or pTKR procedure. This retrospective study was approved by the Human Research Ethics Committee of our institution (RAH HREC Protocol No. 010310a). All data/code/materials relevant to the study are included in the article or are available as supplementary files.

## Results

### Over time administrative and staff changes

The Orthopaedics department at the RAH has undergone several administrative changes between 2003 and 2023 with potential direct effects on its ability to both service delivery and training. Despite a decrease in orthopaedic beds from 96 to 40, an average of 50–60 orthopaedic inpatients is encountered every day. The elective theatre time has increased from 56 hours/week to 63 hours/week while the emergency theatre time has increased from 96 hours/week to 141.5 hours/week. Regarding staff, the number of consultant surgeons FTE has remained unchanged at 8.26 FTE. The number of Fellows has increased from 2 (1 trauma and 1 joint replacement) to 3 (2 trauma, 1 joint replacement), while the number of accredited (Trainee) Registrars has decreased from 6 to 4. The number of unaccredited (Service) Registrars has increased from 3 to 8. Regarding the Service Registrar numbers, it is worthwhile pointing out that due to the decrease in emergency department beds and ramping, 2 Service Registrars are rostered each day on a 12-hour shift to cover the emergency department and provide telephone General Practioner (GP) support, receiving an average 100 telephone calls from GPs/day, while a third Registrar is always rostered off after a night shift. 2 Service Registrar positions were created to replace 2 surgical Resident Medical Officer (RMO) positions to help with the on-call roster that involves a night shift. This in turn has decreased the number of junior medical staff and increased the strain on interns and RMOs.

### Overtime service delivery changes: primary joint replacement

The median waiting time to be seen in the Orthopaedic Outpatient Clinic at Royal Adelaide Hospital (RAH) was reported in the SA Health census to be 12 months (range 0–55 months).^22^ This, of course, includes all acute fractures and other conditions that require urgent attention, which significantly reduces the median waiting time reported. To evaluate the effect of waiting time, from GP referral to surgery, on the deterioration of patients referred for a pTHR and pTKR, we initiated a prospective programme where patients undergo sequential instrumented gait analysis assessments during the waiting period. Preliminary findings demonstrate that patients referred for a pTHR or pTKR wait on average 2 years to be seen in clinic and an additional 1 year to be operated on (Table 1).

**Table 1.** The mean waiting time in days for THR and TKR patients from GP referral to surgery for patients seen in clinic from November 2023 to March 2025.

	GP to surgery (*n =* 26)	GP to clinic (*n =* 38)	Clinic to surgery (*n =* 22)
Max	1931	1773	688
Min	361	35	46
Median	949.5	583.5	404.5
Mean	987.4	715.1	378.9
SD	438.7	508.2	190.5

SD, standard deviation.

Over the last 20 years the number of elective pTHR performed at the RAH has approximately halved from its peak in 2011 (Figure 1(a)). Notably, there has been an increase in the number of patients undergoing a pTHR as an emergency procedure, as more patients with an osteoporotic neck of femur or acetabular fracture now undergo a pTHR. This decrease in number of elective pTHR and pTKR performed has unfortunately been associated with an increase in complexity of the cases and their severity to the extent that of all pTHR and pTKR performed at the RAH in 2024 only 6 were listed as a Category 3 elective case. This has impacted each consultant surgeon, for example, for the surgeon who had most elective theatre time (1 full day operating elective list/week) only 20 primary joints (17 pTHR and 3 pTKR) were performed on all his lists in 2024. None of these cases were ‘regular osteoarthritic’ joints and the various complexity encountered in these cases is illustrated in Figure 3. Simultaneously, the total number of patients awaiting their elective surgery has increased in the last 25 years from 220 in 1999 (pTHR 18) to 597 in 2024 (pTHR 69). In addition to the decrease in exposure of trainees to primary joint replacement surgery, and especially to treat ‘standard osteoarthritic’ joints, we found that during a 6-month rotation, 2 junior trainees and 8 unaccredited registrars performed on average 5.9 hip hemiarthroplasties/rotation (1 hip hemiarthroplasty/month) each.

**Figure 3.**
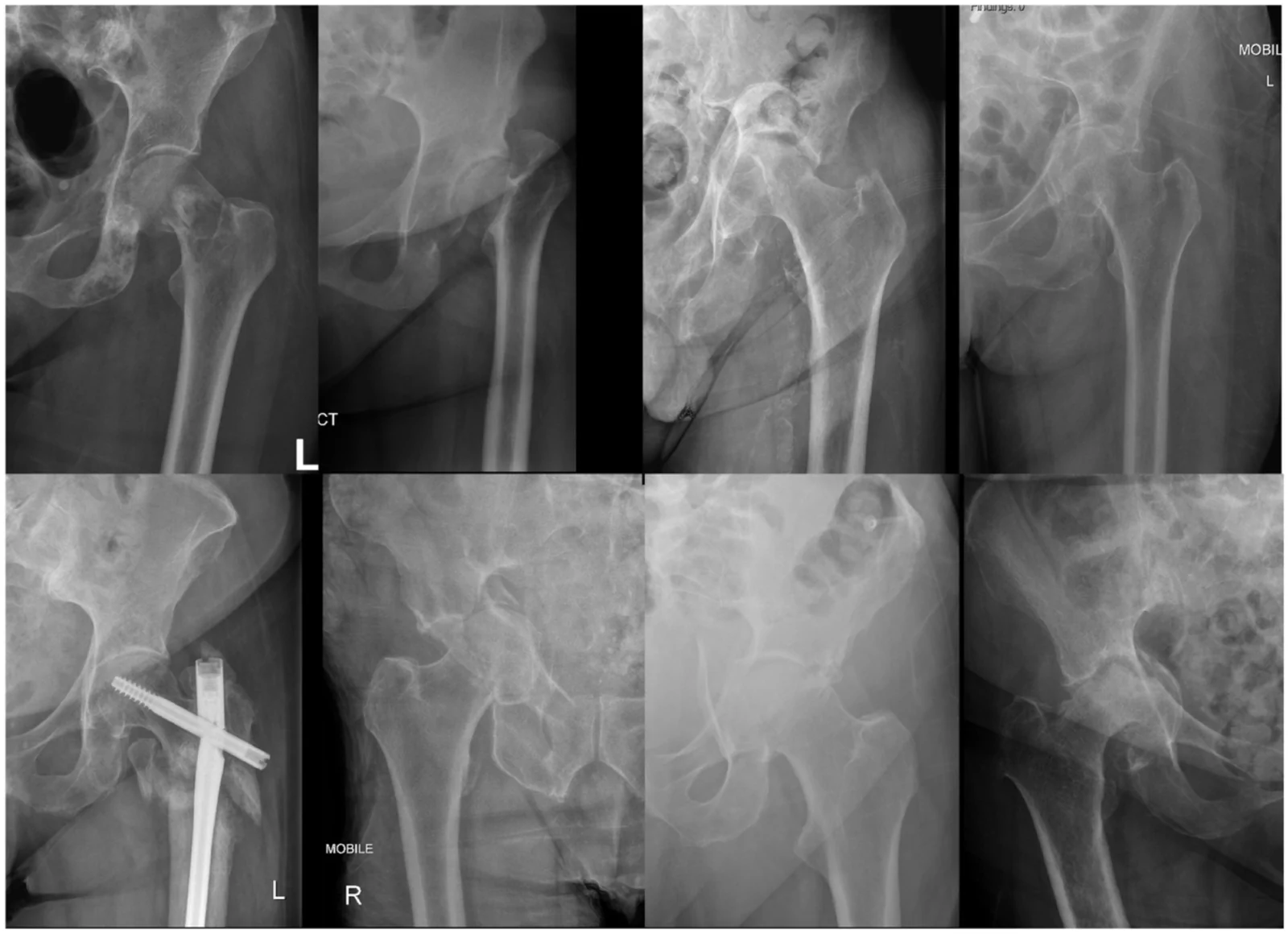
The complexity encountered in primary THR cases performed by 1 surgeon in 2024.

### Revision joint replacement

The number of rTHR surgery performed at the RAH has increased significantly in the last 20 years, due to the need to perform this surgery as an emergency, i.e. due to a PJI or a periprosthetic fracture (Figure 2(b)). There has been a 189% increase in the number of rTHR and rTKRs performed, from 76 in 2003 to 219 in 2023. This increase was almost solely caused by the increase in the number of revisions performed to treat PJI (Figure 4(a)) Regarding rTHR and rTKR performed for aseptic conditions, most of these are now performed for a femoral periprosthetic fracture. In addition to the increase in revisions for periprosthetic fracture, there was also an 583% increase in the number of periprosthetic fractures treated with open reduction internal fixation (ORIF) (considered a reoperation not a revision), from 6 to 41 procedures per year (*p* < 0.001) (Figure 4(b)).

**Figure 4.**
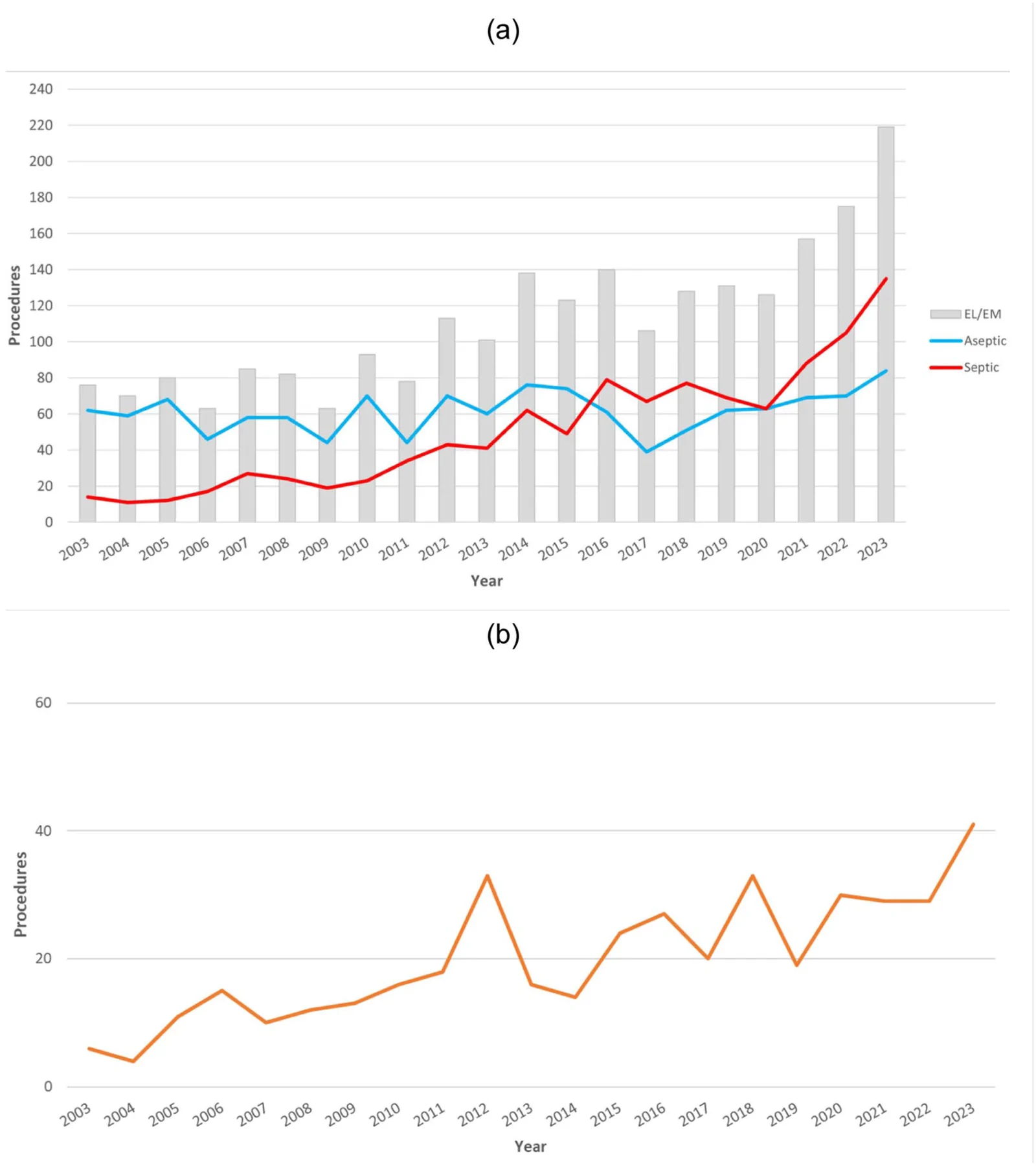
(a) The number of revision THR and TKR performed at the RAH to treat septic and aseptic conditions since 2003. (b) The number of femoral periprosthetic fractures treated with open reduction internal fixation at the RAH since 2003.

The increases in the number of revisions for PJI occurred in both THR and TKR, and reflected the national increases in these procedures reported by the AOANJRR (Figure 5).^2^ This increase in PJI cases managed at the RAH raised questions about where these cases had their index primary procedure and what the in-house infection rate for primary joint replacement was. Of 375 pTHR and pTKR performed at the RAH in 2022 and 2023, to allow for a minimum 1 year of follow-up; the incidence of revision for PJI was 1.6%, which is relatively low given the complexities and the comorbidities of patients treated. During the same 2 years, 152 individual patient rTHR and rTKR were performed to treat PJI at the RAH, with the vast majority (82%) being cases referred from other centres (28 RAH (18%); Private 84 (55%); Other SA Public Hospitals 22 (14%); Interstate/Overseas 17 (11%)), often with previous failed management of the PJI in those centres. Despite this increasing workload, the management of PJI cases at the RAH is in line with recommendations that better outcomes are achieved if PJI cases are managed in specialised referral centres.^15–17^ The RAH has employed an MDT approach for managing PJI for over 20 years which, in combination with a consistent and standardised surgical approach by a specialised team, has enabled cure of PJI in up to 100% of cases at 10 years of follow-up.^13^

**Figure 5.**
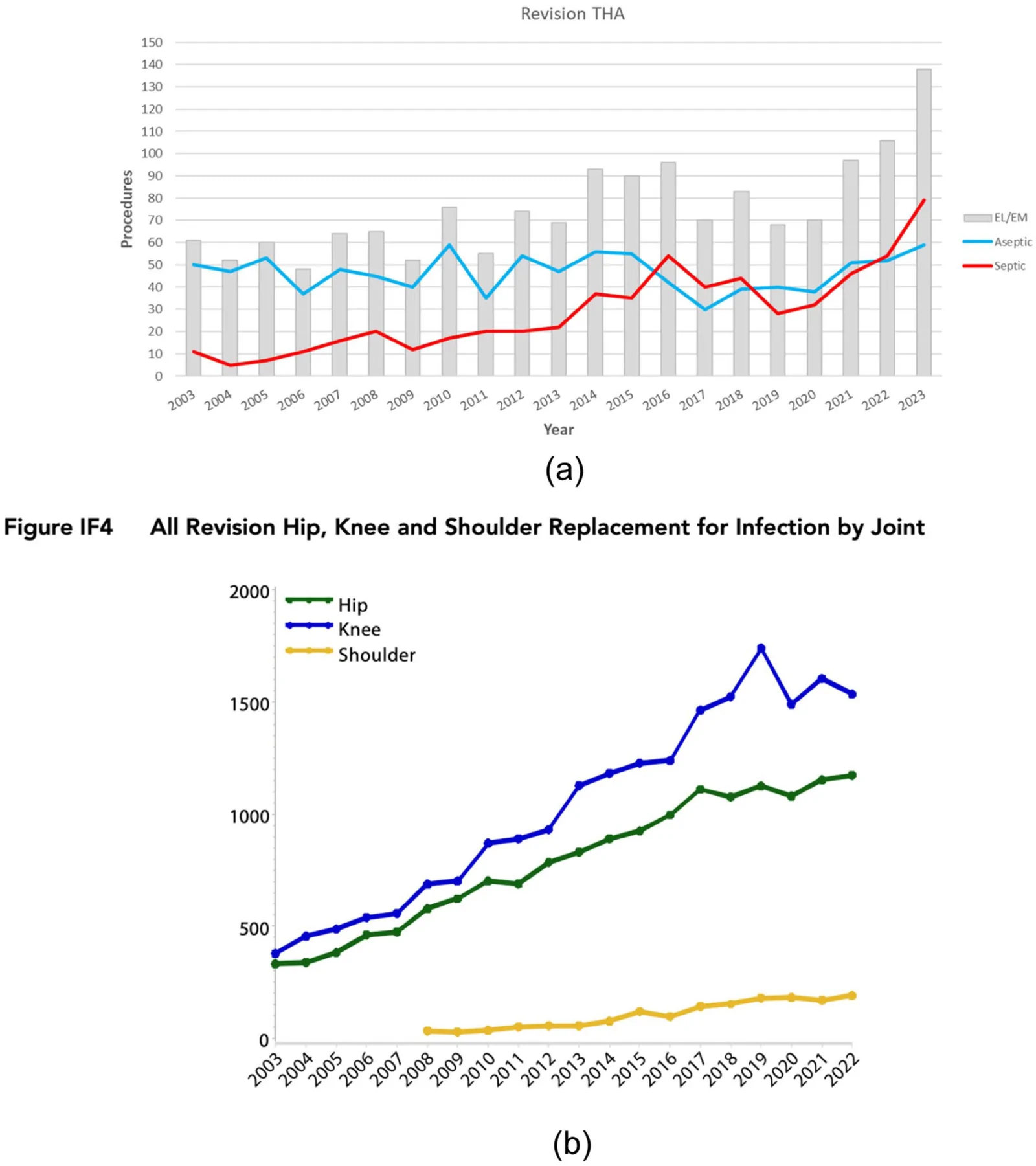
(a) Revisions by reason hip replacements at the RAH. (b) Revisions for infection in the AOANJRR 2023 report.^11^

## Discussion

Over the last 20 years, our public tertiary referral centre has experienced a marked increase in urgent revision joint replacement workload, in addition to an increased complexity of primary elective joint replacement cases, despite a decrease in the number of orthopaedic beds in the hospital. The increase in complexity of cases is invariably associated with an increase in theatre time, as well as length of stay. The increase in theatre time of complex cases will have a flow-on effect of some emergency cases having delayed access to theatre which in turn can increase the length of stay of ‘simple’ cases as their surgery is delayed. This, in association with the decrease in the number of allocated Unit beds, has led to a bed blockage for ‘cold’ elective cases in the absence of beds being ‘ringfenced’ for elective cases. This is compounded by elective orthopaedic cases being generally first in line to be cancelled due to a lack of beds, because they are seen as least urgent by the hospital administrators. The effect of this is reflected in only 6 Category 3 primary THR and TKR being performed in 2024 in what is the largest hospital in the State, which is particularly distressing for a teaching hospital. The increase in case complexity also favours senior trainees/Fellows and reduces the exposure of junior trainees and unaccredited Registrars to ‘simple’ joint replacement cases, compounded by repeat cancellations of non-urgent elective lists. Reduced surgical exposure is particularly concerning when combined with low numbers of simpler hemiarthroplasties for a fracture neck of femur, which is one of the first surgeries Registrars learn, and that the RAH manages the fourth-most neck of femur fractures in Australia.^23^ This in time will undoubtedly have a long-term effect on the younger generation of surgeons, as they will require additional training in the future to reach the equivalent experience level that previous generations of surgeons reached during their formal training.

Waiting times reported often exclude the wait from GP referral to outpatient clinic booking, which accounted on average for 2 years alone in our preliminary analysis. The flow-on effect on the patients’ well-being after the long wait for treatment and not infrequent delays to surgery, is not only physical, with a large percentage of patients suffering from secondary mental health issues.^21^ Importantly, the level of inactivity patients’ reach before undergoing a joint replacement persists after surgery, despite the improvements in pain joint biomechanics and patient-reported outcome measures (PROMS).^24^

As suggested by the AOANJRR data, the situation at our institution is not isolated. As this paper was recently presented at the Arthroplasty Society of Australia annual scientific meeting,^25^ it became obvious that the majority, if not all, of the orthopaedic departments in the major teaching hospitals in all Australian states face the same change in pathology and similar challenges.

Another important effect of the change in pathology at the RAH, and other major Australian public hospitals, is its direct effect on their funding. Treating PJIs is very expensive;^18^ a recent meta-analysis of the cost of revision THR showed that revisions for PJI cost almost double than those for aseptic causes.^26^ Under the AR-DRG classification system; rTHR for dislocation, loosening, fracture and PJI diagnoses all fall under the single I31 code for “revision of hip replacement”,^27^ and therefore public hospitals in Australia are reimbursed the same for any revision irrespective of the reason or what was done. Despite coding shortcomings, the substantial hospital costs of rTHR varies by diagnosis and treatment type.^26^ Analysis of the costs of rTHR at RAH in the last decade revealed that rTHR for dislocation, loosening, periprosthetic fracture and PJI, was associated with a 1.5, 1.9, 2.1 and 2.65 times greater cost than pTHR (AU$25,615) per episode of care respectively.^18,26^ Additionally, surgical treatment of PJI alone varies, with the main surgical treatment types including DAIR, single-stage rTHA and 2-stage rTHA.^28^ If DAIR or revision for PJI fail, as it has been shown to do not infrequently,^28^ ongoing surgical treatment beyond the initial attempt leads to lengthy and complex treatment pathways and, inevitably, to increased costs. Indeed, PJI cases that had 2-stage revision surgery and required re-revisions at the RAH in the last decade had average hospital costs of AU$262,795 and ranged up to AU$508,834.^18^ For RAH patients with the most complex treatment pathways, multiple sequential treatments and readmissions, cumulative hospital costs ranged between AU$272,262 and AU$2,033,724.^29^ Discrepancies between reimbursement and costs of utilising healthcare resources disincentivises healthcare servicing and treating of complicated patient cases in the long-term. This was highlighted in the United Kingdom, where the treatment of fracture patients in a tertiary referral centre incurred a median net loss of £3,757.50/patient.^30^ Therefore, in Australia, specialist centres treating a higher proportion PJI with revision THR are also likely suffering from unexpected reimbursement deficits.

## Conclusion

Key decisions are urgently required at both State and National level to plan for the continual increase in the demand for pTHR and pTKR, as well as the increase in revision joint replacement at tertiary referral centres. Such decisions need to factor in the increasing number of complications of THR and TKR that present as an emergency and make planning more difficult. Without these, treating PJI at specialist tertiary referral centres, which was shown to provide the best results, risks escalating and quickly becoming unsustainable, despite ever increasing efforts to improve diagnosis and optimise treatment strategies. Policy makers are continually required to make important decisions on outsourcing and late cancellation of elective surgeries, including joint replacement, that have a broad impact on the orthopaedic service delivery in public hospitals. In addition, given the importance of advanced surgical training and of retaining trainee Registrars in public hospitals, both for their training and then as young specialist surgeons, they must be given the opportunity to learn and practice primary joint replacement surgery in simpler osteoarthritic cases. If this is not resolved, it will risk not only affecting training but in the longer-term affect service delivery through the negative feedback effect on Registrars and young specialists working in Australian hospitals. The significant change in pathology experience also requires adjustment to staffing, with adequate increases needed at both training and specialist surgeon level. Finally, current hospital reimbursement methods may not adequately account for the complexity of septic revisions compared to aseptic diagnoses, which are currently all accounted for within one AR-DRG code - further categories are required to better reflect actual hospital costs incurred.
